# Plant-Based Culinary Medicine Intervention Improves Cooking Behaviors, Diet Quality, and Skin Carotenoid Status in Adults at Risk of Heart Disease Participating in a Randomized Crossover Trial †

**DOI:** 10.3390/nu17071132

**Published:** 2025-03-25

**Authors:** Andrea M. Krenek, Monica Aggarwal, Stephanie T. Chung, Amber B. Courville, Juen Guo, Anne Mathews

**Affiliations:** 1 Food Science and Human Nutrition Department, University of Florida, Gainesville, FL 32611, USA; akrenek@stanford.edu; 2 National Institute of Diabetes and Digestive and Kidney Diseases, National Institutes of Health, Bethesda, MD 20892, USA; stephanie.chung@nih.gov (S.T.C.); amber.courville@nih.gov (A.B.C.); juen.guo@nih.gov (J.G.); 3 Division of Cardiovascular Medicine, University of Florida, Gainesville, FL 32611, USA; monica.aggarwal@medicine.ufl.edu

**Keywords:** culinary medicine, teaching kitchens, diet quality, plant-based diet, cardiovascular disease, skin carotenoid status, behavior change

## Abstract

**Background**: Culinary medicine (CM) interventions in teaching kitchens have emerged as novel approaches for influencing dietary behaviors, but their efficacy, content, and delivery vary. **Objective**: The effects of a virtual vegan CM intervention on behavioral determinants, cooking competencies, diet quality, and skin carotenoid status were assessed. **Methods**: This analysis from a 9-week randomized crossover study evaluated behavioral survey assessments, Whole Plant Food Density (WPFD) as a diet quality indicator utilizing Automated Self-Administered 24 h Dietary Recall data, and skin carotenoid status (SCS) via pressure-mediated reflection spectroscopy at multiple timepoints. Adults at ≥5% atherosclerotic cardiovascular disease (ASCVD) risk followed a vegan diet pattern that was high or low in extra virgin olive oil (EVOO) for 4 weeks each with weekly virtual cooking classes, separated by a 1-week washout period. Qualitative feedback was collected for thematic analysis. **Results**: In 40 participants (75% female; body mass index, 32 ± 7 kg/m^2^; age, 64 ± 9 years mean ± SD), perceived control over trajectory of heart disease, knowledge of lifestyle behaviors for heart health, and confidence in cooking skills and preparing a variety of plant-based foods improved post intervention (all *p* ≤ 0.001). WPFD increased by 69–118% from baseline. Greater SCS changes occurred after high-EVOO (+51.4 ± 13.9 mean ± SEM, *p* < 0.001) compared to low-EVOO (+6.0 ± 16.4, *p* = 0.718) diets. **Conclusions**: A virtual vegan CM intervention improved dietary behaviors and quality, which was associated with reductions in CVD risk factors. SCS is influenced by EVOO intake, warranting consideration when used to estimate fruit and vegetable intake. The potential impacts of CM on behaviors and health outcomes warrant continued research efforts in medical and public health settings.

## 1. Introduction

The leading causes of death and disease for adults in the US are directly related to lifestyle behaviors, including poor diet, physical inactivity, stress, inadequate sleep, smoking, and excess alcohol consumption [1,2]. While lifestyle interventions support noncommunicable disease prevention [3], effectively implementing behavior changes such as diet intake continues to challenge improvements in the health status of many Americans. Culinary medicine (CM)/culinary nutrition (CN) applied in teaching kitchens (TKs) have emerged as potential methods of influencing dietary and lifestyle behaviors for chronic disease management and prevention through impacting behavioral determinants and change that affect risk factors and health promotion [4].

CM encompasses the evidence-based field that blends the art of preparing, cooking, and presenting food with the science of medicine to target the interconnected underlying metabolic processes shared by several disease states [4,5]. Similarly, CN extends culinary arts and applied nutrition science to various other disease-free populations for general health, performance, or disease prevention [6,7]. CM/CN aim to support the sustainability of nutritional interventions by maintaining sensory enjoyment and positive emotions around food while building cooking skills and confidence.

One form of executing CM/CN is through TKs, which have been described as “learning laboratories for life skills” in a variety of clinical, academic, corporate, community, and in-home settings [8]. Education and hands-on activities in TKs extend beyond dietary intake into additional lifestyle skills. In 2017, Eisenberg et al. reported favorable preliminary changes in health behaviors and clinical biomarkers, including the weight, waist circumference, systolic and diastolic blood pressure, and cholesterol levels of worksite employees through the completion of a prototype TK self-care intervention that integrated mindfulness training and health coaching [9]. This combination of nutrition and lifestyle education with the experiential hands-on learning of cooking skills has been incorporated in TK shared medical appointments, an avenue where patients can learn about and discuss health as a group [10]. TK programming has successfully begun to expand globally, including in Germany [11], Japan [12], Canada [13], Italy, and Israel [14]. However, transportation, time constraints, and access to specialized centers may present challenges. Alternatively, remote culinary coaching programs may offer flexibility and increase engagement, though only a few small-scale studies have explored this avenue [15,16,17,18].

Dietary advice and culinary skills incorporated in CM/CN programming vary based on the setting and purpose. This individualization may include the application of behavioral techniques to implement medical nutrition therapy specific to a disease state, such as cardiovascular disease (CVD) [19], diabetes [17,20], or kidney disease [21], or general public health guidelines for adults or pediatric populations [22]. CM/CN may also be applied to a research diet intervention or activities unique to an individual through personalized lifestyle counseling and culinary coaching. Compared to traditional nutrition counseling, adherence to health-supportive dietary patterns may improve through the use of CM/CN [23]. Moreover, CM/CN aim to support improvements in diet quality, which has long been considered a strong predictor of morbidity and mortality [24,25,26,27,28,29]. Relationships between diet intake and disease can be assessed by diet quality indices (DQIs), which are assessment tools used to evaluate the extent to which intake aligns with a specified pattern. In the context of CVD, plant-based patterns, such as vegan/vegetarian diets, and an emphasis on whole foods are advised for risk reduction [30,31,32]. One DQI evaluating plant food intake is Whole Plant Food Density (WPFD), which has been evaluated for assessing target food groups in behavioral nutrition interventions [33,34,35,36]. In conjunction with the evaluation of diet quality, which is often calculated from subjective methods of diet assessment, research efforts are devoted towards identifying simple and objective modes of capturing diet intake [37,38]. Skin carotenoid status (SCS) is an emerging noninvasive proxy for fruit and vegetable (F/V) intake as a key component of a healthful dietary pattern [39,40].

Evidence on these applications, diet and behavioral effects, and best practices for culinary medicine programming [41] is promising but limited. Further, testing a culinary-based vegan diet and multimodal lifestyle education intervention in a virtual setting has not been previously reported. Therefore, we assessed the efficacy of a virtual TK to deliver a plant-based CM curriculum during the Recipe for Heart Health study, a randomized crossover clinical trial evaluating high versus low extra virgin olive oil intake [42] in adults ranging in age and weight classification. As expected, significant improvements in cardiometabolic risk profiles were seen in response to both diet interventions compared to baseline intake. Thus, this pre-planned secondary outcome analysis investigates the cumulative effect of the CM intervention on behavioral determinants, cooking competences, diet quality, and skin carotenoid status. We hypothesized that the vegan CM intervention would enhance the adoption of a healthful dietary pattern as well as improve cooking-related competencies and skin carotenoid status.

## 2. Materials and Methods

This study protocol was approved by the University of Florida Institutional Review Board (IRB202002194, approved 4 September 2021), and all study participants provided written informed consent obtained by investigators or trained study coordinators prior to any study procedures.

### 2.1. Study Design and Sample

**Figure 1:**
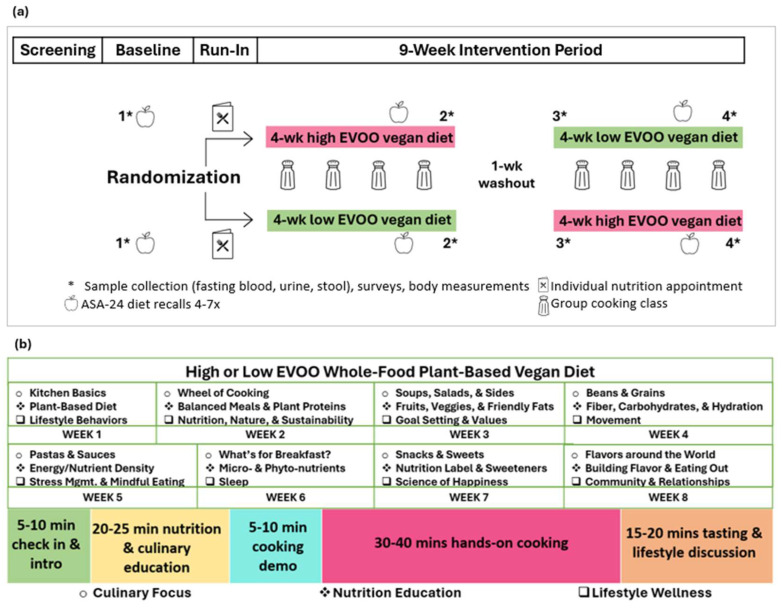
Recipe for Heart Health study design (**a**) and cooking class content curriculum overview (**b**). Abbreviations: EVOO, extra virgin olive oil; ASA-24, Automated Self-Administered 24 h Dietary Assessment Tool. Circle, diamond, and square symbols indicate the culinary focus, nutrition education, and lifestyle wellness topic of the teaching kitchen curriculum.

Recipe for Heart Health was a randomized crossover clinical trial (NCT04828447) investigating high (4 tablespoons/day) vs. low (<1 teaspoon/day) intake of extra virgin olive oil (EVOO) within a whole-food plant-based vegan diet. Adults (18–79 years) with ≥5% atherosclerotic cardiovascular disease (ASCVD) risk according to the ACC/AHA ASCVD risk calculator were recruited from medical clinics in Gainesville, Florida, within the University of Florida Health network starting in April 2021 with final data collection concluding in May 2022. Secondary prevention patients, pregnant individuals, those already following a Mediterranean or vegan diet, warfarin use, and conditions that would impact study outcomes outside of the intervention were excluded (detailed exclusionary criteria published previously [42]). Participants recruited via approved electronic medical record portals and at patient visits were randomized using computer-generated sequencing to follow both diet patterns in a random order for four weeks each (Figure 1).

### 2.2. Virtual Culinary Medicine Teaching Kitchen Intervention

Outlined in Figure 1, virtual cooking classes were facilitated by a dietitian/chef through Zoom™ weekly during both 4-week diet intervention periods for a total of eight approximately 90 min sessions. In addition to individual guidance and meal planning on following the vegan diet, participants were advised on oil-free cooking methods to maintain during the low-EVOO diet period and to add EVOO to meals during the high-EVOO diet period. All EVOO was provided over the high-EVOO study period in addition to weekly gift cards to support grocery purchases. Each virtual class included a focused discussion on culinary skills, nutrition education, and lifestyle behaviors during sessions that integrated didactics with interactive cooking and group support. General class structure included approximately 30 min of check-in communication on diet changes, nutrition and culinary education, and brief cooking demonstrations, followed by 30–40 min of hands-on cooking and 15–20 min of further discussion around other lifestyle topics while tasting the meals that were prepared (Figure 1). A “study cookbook” provided to participants contained recipe options that aligned with the diet interventions and the culinary focus of the week for participants to choose from to prepare during the virtual class. Along with individual email recipe links, the study cookbook included ~15–40 recipe option ideas for each session, culminating in exposure to ≥200 recipes in addition to adaptable meal templates. Progress pages to note challenges, successes, questions, planning, and goals as well as other lifestyle behavior resources related to the lifestyle behavior wellness topic of the week were also included in the study cookbook. Additional supporting resources related to class topics were included in weekly pre- and post-class emails. Participants were provided with recommendations for optional videos, articles, podcast episodes, Facebook group engagement, and online cooking platform sites. The last cooking class further applied concepts integrated throughout the intervention as participants selected and adapted ingredients as needed to prepare a recipe of their own choosing.

### 2.3. Outcomes

Medication history and sociodemographic data, including age, sex, cooking and grocery shopping habits, race/ethnicity, education and income status, and children in household were collected at baseline. In the current analysis, medication history is presented as descriptive background, which may affect outcomes; reported treatment of hypertension, statin therapy, and aspirin use is considered in the ACC/AHA ASCVD risk calculator.

Behavioral and dietary measures were assessed at time points before and after the two 4-week diet intervention periods. Five to seven days of 24 h diet recalls were collected at baseline and the last week of each diet period via the National Cancer Institute’s Automated Self-Administered 24 h (ASA-24) Dietary Assessment Tool [43] to evaluate adherence. This multi-pass method is a freely available web-based tool used to capture nutrient intake over a full 24 h period. Participants were advised to include both week and weekend days. Compliance was additionally affirmed through weekly verbal check-ins during group cooking classes. As previously published [42], reported nutrients and food groups confirmed dietary adherence to the whole-food plant-based vegan diet, including avoidance of animal foods. As another indicator of animal food intake that reflected reported values, trimethylamine N-oxide (TMAO) was additionally measured by liquid chromatography with tandem mass spectrometry through Cleveland HeartLab (Cleveland, OH, USA) [44]. EVOO reported through ASA-24 recalls reflected prescribed values for each group (mean + SD 3.74 + 0.54 TB during high intake of EVOO vs. 0.03 + 0.15 tsp during low intake of EVOO). Based on diet recalls, diet quality was assessed by the WPFD as the sum of cup or ounce equivalents per 1000 kcal of whole grains, legumes, whole fruit, vegetables, and nuts and seeds [33]. To assess self-reported changes in fruit and vegetable intake and evaluate differences in carotenoid concentration related to fat intake, skin carotenoid status was measured by the pressure-mediated reflection spectroscopy-based VeggieMeter^®^ (Longevity Link, Corp, Salt Lake City, UT, USA) reflection spectroscopy [39,45].

Cooking and food agency was measured using the Cooking and Food Provisioning Action Scale (CAFPAS) [46], a 28-item measure that can be divided into three subscales: self-efficacy on abilities and skills around cooking, attitude related to views towards food and cooking, and structure to assess external factors that can influence cooking actions and goals. Survey items were rated on a 7-point Likert scale, with an overall higher CAFPAS score indicating higher cooking and food agency that correlates to predicted reported meals cooked per week.

To capture cooking skills and confidence preparing a variety of plant-based foods, participants responded to questions adapted from validated measures employed in similar interventions on a 5-point Likert scale (1 = not confident at all, 5 = completely confident) [47,48,49,50]. Based on a similar teaching kitchen randomized controlled trial, composite scores for this survey were calculated as the average of the 10 and 20 items, respectively.

Participants also rated their agreement (1 = strongly disagree, 5 = strongly agree) on six custom items developed by the research team to assess knowledge of plant-based diets and lifestyle behaviors for heart health, perceived benefits, and perceived control over heart disease. To assess satisfaction and qualitative feedback on cooking classes, brief weekly surveys were sent to participants to complete online. Survey questions assessed their satisfaction with each of the eight cooking classes, their satisfaction with recipe options provided each session, what could make live cooking sessions more helpful, and broad additional open-ended sharing of any other thoughts. Two reviewers who did not participate in study design, cooking classes, or data collection independently evaluated comments for thematic analyses. This qualitative feedback on weekly cooking classes was informally used to help improve sessions and inform future investigations. The results of metabolic and clinical data have been previously published [42].

### 2.4. Statistical Analysis

Participant baseline characteristics are presented as means ± SDs or percentages. Inferential numerical outcomes are reported as means ± SEM in the text and tables. Statistical analyses were performed using R version 4.1.3 and SAS 9.4. All statistical tests were two-sided, with significance considered at the *p* < 0.05 level. Normality was visually assessed by histograms/QQplots in addition to performing Shapiro–Wilk tests. Linear mixed models analyzed SCS changes between diets, including age, sex, and body weight change as covariates. Pre–post-intraindividual SCS and scores for cooking and food agency, reported cooking skills and confidence, diet quality, and beliefs and knowledge related to nutrition and heart health were analyzed by a paired *t*-tests (parametric) or Wilcoxon Signed-Rank tests (nonparametric). Sample size determination was based on power calculations for the primary outcome, low-density lipoprotein cholesterol, reported elsewhere [42]. In summary, to detect a mean difference in low-density lipoprotein cholesterol of 0.30 mmol/L (12 mg/dL) with a SD ± 20 mg/dL, and the assumption that within-subject level correlations were 0.3, a total of 40 participants would yield a power of 0.8 and a type 1 error probability of 0.05.

## 3. Results

Forty participants (mean ± SD 64.4 ± 8.6 years, including adults between 36 and 78 years) enrolled in the study and completed behavioral and dietary measurements. Supplemental Figure S1 summarizes recruitment and enrollment. Most participants were female (75%), had obtained at least a college degree (65%), and were the primary person responsible for grocery shopping (92.5%) and cooking meals (80%) in the household, Table 1. As previously reported, both high- and low-EVOO diets resulted in decreased low-density lipoprotein cholesterol and improved cardiometabolic risk profile compared to baseline that improved to a greater extent during the low-EVOO diet [42]. Three participants expressed experiencing transient uncomfortable digestive symptoms with diet changes.

### 3.1. Knowledge, Perceived Control, and Perceived Benefit of Behavioral Determinants

Knowledge about dietary and lifestyle behaviors for heart health as well as perceived control over one’s trajectory of heart disease significantly increased (+31% and +11%, respectively) from baseline to post intervention (*p* < 0.0001 and *p* = 0.005), Table 2. No significant changes were observed in perceived benefit of plant-based diets for heart disease, for which scores were close to maximum response values at baseline (+10%, *p* = 0.114).

### 3.2. Cooking Competencies

Overall cooking and food agency scores significantly increased from 12.4 ± 0.4 to 15.3 ± 0.4 (*p* < 0.001), driven by an 80% increase in mean self-efficacy scores (+2.5 ± 0.2, *p* < 0.001), Table 2 [51]. Attitude towards and structural barriers of food and cooking did not increase significantly (+0.2 ± 0.2, *p* = 0.203 and +0.2 ± 0.2, *p* = 0.354, respectively). Confidence in cooking skills as well as confidence preparing a variety of plant-based foods both increased compared to baseline (*p* ≤ 0.001).

### 3.3. Diet Quality

Comparison of diet quality indicated significantly greater intake of whole plant foods as a composite index of WPFD from 2.9 ± 1.5 cup/oz-equivalents per 1000 kcal pre-intervention to 5.0 ± 1.4 and 6.4 ± 2.0 cup/oz-equivalents per 1000 kcal during the high- and low-EVOO phases, respectively (*p* < 0.0001, Table 3) [52]. Greater energy intake consumed during the high EVOO period appears to indicate a lower WPFD when standardized per 1000 kcal, though absolute values are comparable. For both diets, each subcomponent including whole grains, legumes, fruits, vegetables, and nuts and seeds significantly increased from baseline, with no differences detected between diets. No differences in food acceptability were detected after the high-EVOO and low-EVOO periods. Overall acceptability of either pattern was high. Participants indicated slightly greater effort to adhere to high compared to low amounts of EVOO, though paradoxically predicted a slightly increased ability to remain on the high daily EVOO diet more of the time.

### 3.4. Skin Carotenoid Status

**Figure 2:**
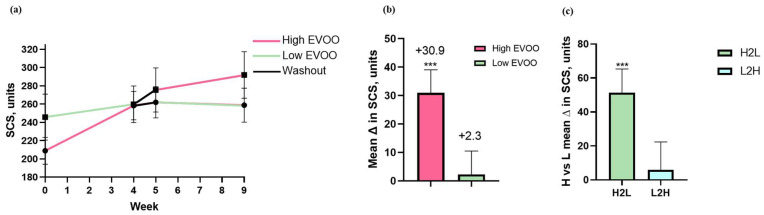
Skin carotenoid status (SCS) levels at each timepoint before and after the high- and low-EVOO vegan diets (**a**). Change in skin carotenoid status from baseline comparing the high- and low-EVOO vegan diets by both diet periods (**b**) and by sequence of diet randomization (**c**). Data are mean + SEM. The difference between the high- and low-EVOO groups in SCS changes from baseline was analyzed by a linear mixed model adjusted for age, sex, and body weight change. *** *p* < 0.001. Abbreviations: EVOO, extra virgin olive oil; H2L, high to low EVOO diet order; L2H, low to high EVOO diet order; SCS, skin carotenoid status.

While participants’ SCS increased during both the high- and low-EVOO vegan diet periods, SCS increased more following the high-EVOO period (+30.9, *p* < 0.001) [53]. The difference between diets was 28.7 ± 10.5, *p* = 0.009. Considering an order effect (*p* = 0.045), greater SCS changes occurred during the high-EVOO diet (+51.4 ± 13.9, *p* < 0.001) compared to the low-EVOO diet (+6.0 ± 16.4, *p* = 0.718), in addition to differences in SCS between the high- and low-EVOO diets considering all four timepoints, Figure 2. Relevant to SCS measurements, self-reported daily fruit and vegetable intake was 2.1 ± 1.5 and 1.0 ± 0.7 cups, respectively, at baseline and each increased by approximately 0.5–1 cups with no differences between diets. During the high- and low-EVOO diets, fat consumption comprised 48% and 32% of energy, respectively.

### 3.5. Thematic Analysis of Participant Feedback on Cooking Classes

Reported cooking class ratings as ranked on a 1 to 10 Likert scale ranged from averaged scores of 8.8 to 9.7, with all except one class reporting above a mean score of 9 out of 10. The highest rated cooking class was week 4, which covered the preparation of beans and grains, nutrition education on dietary fiber, carbohydrates, and hydration, and a lifestyle discussion on physical activity. Week 2, which discussed cooking methods, plant proteins, nature–health connection, and sustainability had the lowest reported score. Nine themes identified from qualitative participant feedback from each cooking class included educational content, views on delivery mode, impact on lifestyle behaviors and knowledge, and support, Table 4.

## 4. Discussion

This investigation demonstrated the beneficial effects of a vegan group culinary medicine teaching kitchen intervention on cooking behavioral measures, dietary outcomes, and SCS in adults (including young, middle-aged, and older adults with a variety of weight status classifications) at risk for heart disease (clinical outcomes reported previously [42]).

Culinary-related changes coincided with diet quality improvements, which were indicated by greater intake of whole plant foods. Congruent with our hypotheses, we observed increases after participating in the intervention in (1) reported *knowledge* of dietary and lifestyle behaviors for heart health, (2) *perceived control* over trajectory of heart disease, (3) *confidence in preparing* a variety of plant-based foods, (4) *confidence in cooking* skills, and (5) cooking and food *agency*. These measures all fall within key constructs of Social Cognitive Theory [54] and Self Determination Theory [55] frameworks as determinants of behavior change. As these frameworks outline personal, behavioral, and environmental factors which may be affected through cooking, these behavioral frameworks may be particularly useful for evaluating culinary interventions. Multiple investigations of culinary medicine interventions have similarly reported increased knowledge and confidence around cooking and food behaviors [7]. Cooking and food skills as well as cooking confidence, attitudes, and perceptions contribute towards *food agency*, or one’s ability to procure and prepare food [56]. Food agency may also be described as enhanced empowerment to cook, or to act to set and achieve cooking and food-related goals, for which results suggest improvements among participants. Increased *perceived control* overlaps with cooking and food agency in supporting *confidence* for making autonomous lifestyle choices to manage one’s life and health outcomes. These changes further indicate enhanced ability to create what was envisioned and constructively respond to unanticipated challenges in the kitchen. Reinforced by CAFPAS subscale analyses, self-efficacy is a critical facet of influencing agency. Moreover, food agency may predict meals cooked per week with correlations to the more frequent cooking of meals, reduced use of packaged ingredients, and higher intake of vegetables, which may translate to improved nutrient intake [56].

Cooking more frequently at home is associated with better diet quality and other healthy behaviors, such as physical activity, across socioeconomic backgrounds [57,58]. In this study, we evaluated diet quality by assessing changes in overall WPFD and subcomponents including whole grains, legumes, whole fruit, vegetables, and nut/seeds, which are the foundation of recommended healthy dietary patterns [30,59]. The increased consumption of these whole plant foods contributes towards suggested intake for reducing cardiovascular disease risk, which may be reduced by 27% [60,61] with 3–4 daily servings of fruits, vegetables, and legumes [62]. Increases in whole plant foods mirrored greater dietary fiber intake, a dietary factor utilized in medical nutrition therapy that is well known for reducing chronic disease risk [63,64,65,66].

While the benefits of fruit and vegetable intake for reducing chronic disease risk are recognized, non-invasive and objective assessment of consumption, such as through skin carotenoid levels, is of particular interest to validate and identify levels correlated with improved health more clearly. Analysis of skin carotenoid status interestingly showed higher values during the high-EVOO diet, despite similarly reported F/V intake as during the low-EVOO diet. These differences may have been influenced by fat intake, which is required for carotenoid absorption, concentrated carotenoids in olive oil, and body weight changes through carotenoid storage in adipocytes. As SCS may be influenced by dietary fat, its intake warrants consideration when using SCS to estimate F/V consumption.

Lastly, food acceptability reported after each diet period was overall relatively high and comparable during the high- and low-EVOO vegan diets. While views were generally mixed, several participants expressed a dislike of consuming 4 TB of EVOO in contrast to their usual lower intake, particularly in uncooked form, though any short-term dietary changes may elicit negative responses due to lack of familiarity. A few participants additionally reported digestive discomfort (diarrhea, loose stools) with the immediate increase in EVOO intake.

We recognize limitations of this study in interpreting outcomes. First, because the nonprimary outcomes reported in this paper were not the basis for power calculations, significance should be confirmed in future investigations. Without a control group that did not participate in cooking classes, it is not possible to confirm whether results would differ for an inactive group in this study. With a sample of predominantly highly educated White older adult women who were overweight or obese and who were motivated and interested in cooking (or had willing family members), generalizability may be limited as educational background may affect cooking literacy, motivation for making behavioral health changes, and knowledge of resources. Study strengths include a low attrition rate, complete and comprehensive data collection, defined study objectives, novelty in metabolic data collection in a CM intervention and intervention content, and the use of CM for investigating research questions beyond the TK effects alone.

Cooking confidence and skills, food agency, and diet quality all significantly improved compared to pre-intervention levels. Skin carotenoid status varied by EVOO consumption. Few studies have analyzed metabolic and clinical measures in response to CM programs administered remotely to date, though this mode of administration and published data are rapidly developing as a response to the COVID-19 pandemic. The Recipe for Heart Health study adds novelty through its use of a CM TK for evaluating a vegan diet pattern, supporting adherence in investigating additional research questions, and a group virtual format that integrates multiple lifestyle components. The findings may aid in guiding subsequent behavioral or clinical and community research interventions. The evaluation of longer-term effects in multiple populations in a variety of settings while considering the influence of TK design (e.g., duration and frequency of sessions, curricula content, mode of delivery), individual nutritional factors, and social determinants of health are among areas for further research. Comprehensive well-planned data collection that includes both behavioral and metabolic outcomes is warranted, particularly in the context of developing effective Food is Medicine interventions and programming [67].

## 5. Conclusions

This study evaluated behavioral and dietary outcomes of a multi-modal lifestyle-focused virtual TK intervention in cardiology patients at risk of heart disease. Participation in Recipe for Heart Health led to improvements in nutrition and lifestyle-related knowledge, perceived control over trajectory of heart disease, confidence in food preparation and cooking skills, and food agency. Promoting a plant-forward dietary pattern may further aid in improving dietary quality and skin carotenoid status. As an evidence-based comprehensive and experiential TK curricula, Recipe for Heart Health may serve as a model for informing clinical dietary and lifestyle interventions. With the potential of CM TK avenues to improve lifestyle behavior change and quality of life, additional research with larger-scale implementation is needed to evaluate the effects on health behaviors, clinical risk factors, and psychological well-being.

## Figures and Tables

**Table 1:** Baseline characteristics of Recipe for Heart Health study population by randomization.

Characteristic	All (*n* = 40)	High to Low EVOO (*n* = 22)	Low to High EVOO (*n* = 18)
Age, years	64.4 ± 8.6	65.5 ± 6.3	63.0 ± 10.9
Sex, female, *n* (%)	30 (75%)	14 (64%)	16 (89%)
Primarily responsible for cooking meals, *n* (%)	32 (90%)	17 (77.3%)	15 (83.3%)
Primarily responsible for grocery shopping, *n* (%)	37 (92.5%)	20 (90.9%)	17 (94.4%)
Medication			
Lipid-lowering drug	21 (52.5%)	13 (59.1%)	8 (44.4%)
Antihypertensive	24 (60%)	12 (54.5%)	12 (66.7%)
Aspirin/other antithrombotic	13 (32.5%)	8 (36.4%)	5 (27.8%)
Oral hypoglycemic drug	1 (2.5%)	0 (0%)	1 (5.6%)
Insulin	9 (22.5%)	2 (9.1%)	7 (38.9%)
Previously used tobacco *	12 (30%)	7 (31.8%)	5 (27.8%)
Race/ethnicity, *n* (%)			
African American or Black	9 (22.5%)	5 (22.7%)	4 (22.2%)
Asian or Pacific Islander	1 (2.5%)	0 (0%)	1 (5.5%)
Hispanic/LatinX	1 (2.5%)	1 (4.5%)	0 (0%)
Non-Hispanic White	29 (72.5%)	15 (68.1%)	14 (77.7%)
Other	2 (5.0%)	2 (9.0%)	0 (0%)
Highest level of education achieved, *n* (%)			
High school degree	1 (2.5%)	0 (0%)	1 (5.6%)
Some college	13 (32.5%)	7 (31.8%)	6 (33.3%)
College degree	11 (27.5%)	6 (27.3%)	5 (27.8%)
Some post-graduate degree	2 (5.0%)	1 (4.5%)	1 (5.6%)
Post-graduate degree	13 (32.5%)	8 (36.4%)	5 (27.8%)
Income, *n* (%)			
USD 10,000–25,000	3 (7.5%)	0 (0%)	3 (16.7%)
USD 25,000–50,000	6 (15%)	5 (22.7%)	1 (5.6%)
USD 50,000–75,000	5 (12.5%)	2 (9.1%)	3 (16.7%)
USD 75,000–100,000	7 (17.5%)	3 (13.6%)	4 (22.2%)
USD 100,000–150,000	6 (15%)	5 (22.7%)	1 (5.6%)
USD > 150,000	10 (25%)	7 (31.8%)	3 (16.7%)
Prefer not to answer	3 (7.5%)	0 (0%)	3 (16.7%)
Children in household, *n*	1 (2.5%)	0 (0%)	1 (5.6%)
BMI			
Women	31.8 ± 7.6	31.2 ± 6.8	32.3 ± 8.4
Men	32.3 ± 5.6	33.1 ± 5.9	29.4 ± 4.2
All	32.0 ± 7.1	31.9 ± 6.4	30.2 ± 4.8

Data represent mean ±SD or *n* (%). * No participants reported currently smoking. “Some post-graduate degree” indicates partial but not full completion of post-graduate studies in contrast to the “Post-graduate degree” selection, indicating completion of the degree.

**Table 2:** Summary of changes in cooking outcomes and behavior change determinants.

	Baseline	Post Intervention	Mean Difference	*p*-Value
Confidence of cooking skills	4.1 ± 0.1	4.5 ± 0.1	+0.4 ± 0.1	0.001
Confidence preparing a variety of plant-based foods	3.6 ± 0.1	4.3 ± 0.1	+0.7 ± 0.1	<0.001
Knowledge of lifestyle behaviors for heart health	3.5 ± 0.2	4.6 ± 0.1	+1.1 ± 0.2	<0.001
Perceived control over trajectory of heart disease	3.7 ± 0.1	4.1 ± 0.1	+0.4 ± 0.1	0.003
Perceived benefit of plant-based diets for heart health	4.4 ± 0.1	4.6 ± 0.1	+0.3 ± 0.2	0.096
Cooking and food agency				
Overall	12.4 ± 0.4	15.3 ± 0.4	+2.9 ± 0.4	<0.001
Self-efficacy	4.1 ± 0.2	6.6 ± 0.2	+2.5 ± 0.2	<0.001
Attitude	3.8 ± 0.2	4.0 ± 0.2	+0.2 ± 0.2	0.203
Structure	4.6 ± 0.1	4.5 ± 0.1	+0.2 ± 0.2	0.354

Values are presented as mean ± SEM. Results obtained from paired *t*-tests for baseline to post-intervention comparisons.

**Table 3:** Mean daily intake of Whole Plant Food Density subcomponents and standardized (per 1000 kcal) scores.

	Baseline	High EVOO	*p* Value Baseline vs. High EVOO	Low EVOO	*p* Value Baseline vs. Low EVOO	*p* Value High vs. Low EVOO
Energy, kcal/d	1822 ± 674	1745 ± 513	0.462	1338 ± 374	<0.001	<0.001
Whole grains, oz eq	1.13 ± 0.92	2.20 ± 1.63	<0.001	2.18 ± 1.53	<0.001	0.925
Whole grains, oz eq/1000 kcal	0.66 ± 0.50	1.26 ± 0.68	<0.001	1.61 ± 0.97	<0.001	0.004
Legumes, cup eq	0.09 ± 0.09	0.37 ± 0.27	<0.001	0.35 ± 0.29	<0.001	0.527
Legumes, cup eq/1000 kcal	0.05 ± 0.06	0.22 ± 0.16	<0.001	0.27 ± 0.20	<0.001	0.185
Whole fruit, cup eq	1.03 ± 0.71	1.56 ± 0.93	<0.001	1.48 ± 0.89	0.001	0.466
Whole fruit, cup eq/1000 kcal	0.61 ± 0.46	0.92 ± 0.55	0.002	1.15 ± 0.77	<0.001	0.019
Vegetables, cup eq	2.13 ± 1.48	2.82 ± 1.15	0.001	2.56 ± 1.18	<0.001	0.147
Vegetables, cup eq/1000 kcal	1.18 ± 0.72	1.68 ± 0.74	0.001	1.99 ± 1.06	<0.001	0.014
Nuts/seeds, oz eq	1.94 ± 1.63	1.56 ± 1.22	0.001	1.94 ± 1.63	<0.001	0.242
Nuts/seeds, oz eq/1000 kcal	0.44 ± 0.47	0.87 ± 0.62	<0.001	1.39 ± 1.11	<0.001	<0.001
Whole Plant Food Density	2.93 ± 1.48	4.96 ± 1.37	<0.001	6.41 ± 2.05	<0.001	<0.001

Whole Plant Food Density calculated as the sum of cup or ounce equivalences of whole grains, legumes, vegetables, and nuts/seeds per 1000 kilocalories. *p* values obtained from paired t-tests for each comparison apart from legumes, for which a nonparametric test (Wilcoxon Signed-Rank) was applied. Abbreviations: kcal, kilocalories; d, day; oz eq, ounce equivalents; cup eq, cup equivalents.

**Table 4:** Selected sample comments on cooking classes from Recipe for Heart Health participants.

Theme	Participant Feedback
Educational content	▪ I am learning everything about cooking and food choices from the ground level up… I found the “classroom lectures” very helpful…what kind of knife to use… how to chop… questions and answers… ▪ I’m well pleased in the food education that I have received thus far. It’s a shame that this food education could not have come earlier in my life. I plan on passing all that I can onto my kids (grown) and grandkids. ▪ …we are more than just “doing”, we are learning much more about the “why” and are understanding how it all works together… I’m enjoying the class very much and appreciate all of the help given ▪ …put together a well thought out and comprehensive course. The weekly classes help pull it all together on the topic at hand. I particularly like the follow up to questions, i.e. follow up emails with additional links/videos to more resources
Technology	▪ I’m a little techy challenged. I was unable to see some screens on my tablet. I’ll try a laptop next. ▪ Some problems scanning through recipe book to find what looking for
Impact on lifestyle and knowledge	▪ This study has changed my life!!!… thank you for the direction and motivation that I needed to change my life permanently for the better!! ▪ This has been a life changing experience that we plan to continue ▪ This research study has been a blessing for us ▪ I very much enjoyed being part of this study. It has not only introduced me to different types of foods and cooking, it has changed my eating habits for the better ▪ Keep this study going as long as possible so many people could use this in their life.
Impact on symptoms or behaviors	▪ Have found that a plant-based diet has not left me feeling hungry at any time ▪ I’m more mindful of what I’m eating now. I’m beginning to plan my meals ahead and prep on the weekend for the upcoming week. Thank you. ▪ Really surprised that I haven’t been hungry ▪ I was pleasantly surprised that the food actually tasted so good—I did not feel deprived like I usually do on a “diet”
Recipes	▪ Very satisfied. I enjoyed all the recipes prepared…. and extremely fulfilling ▪ Excellent choices… the variety was great!
General satisfaction	▪ Overall, I love the zoom classes and have already learned a good deal. Thanks! ▪ I am amazed at the variety of foods and how well the program is set up. ▪ Thank you for offering this opportunity… I am very thankful for the classes ▪ This is a really great study. I feel blessed to be a part of it.
Support	▪ Enjoying the input from the rest of the team ▪ All in all, I enjoyed the class very, very much and learned a tremendous amount about myself, and about eating in a whole new way. It was also fun to be in the Zoom classes and to make friends with the others in the class. l hope that we will be able to facilitate a “reunion” after a few months and see how everyone is doing and how they’ve used the diet in their lives. Thank you for having the classes and for including me in them.
Delivery mode	▪ The live sessions were terrific and meeting by Zoom was terrific and fun and made “getting to class” much easier and more workable ▪ Of course, not having to do the cooking sessions on zoom would be good. It would be fun to be able to follow up the cooking sessions with a tasting party in a perfect world!!!
Instructor-specific	Available upon request.

## Data Availability

Data are available from the authors upon reasonable request due to privacy reasons.

## Supplementary Materials

The following supporting information can be downloaded at: https://www.mdpi.com/article/10.3390/nu17071132/s1, Figure S1: Recipe for Heart Health Participant CONSORT Flow Diagram.
