# Supplementary material for: Plant-Based Culinary Medicine Intervention Improves Cooking Behaviors, Diet Quality, and Skin Carotenoid Status in Adults at Risk of Heart Disease Participating in a Randomized Crossover Trial
Source: Nutrients. 2025 Mar 25;17(7):1132. doi: 10.3390/nu17071132 (PMC11990422; doi:10.3390/nu17071132)
Supplement: Supplementary file 1 [file nutrients-17-01132-s001.zip › nutrients-3522952-supplementary.pdf]

SUPPLEMENTARY MATERIAL

Figure S1: Recipe for Heart Health Participant CONSORT Flow Diagram

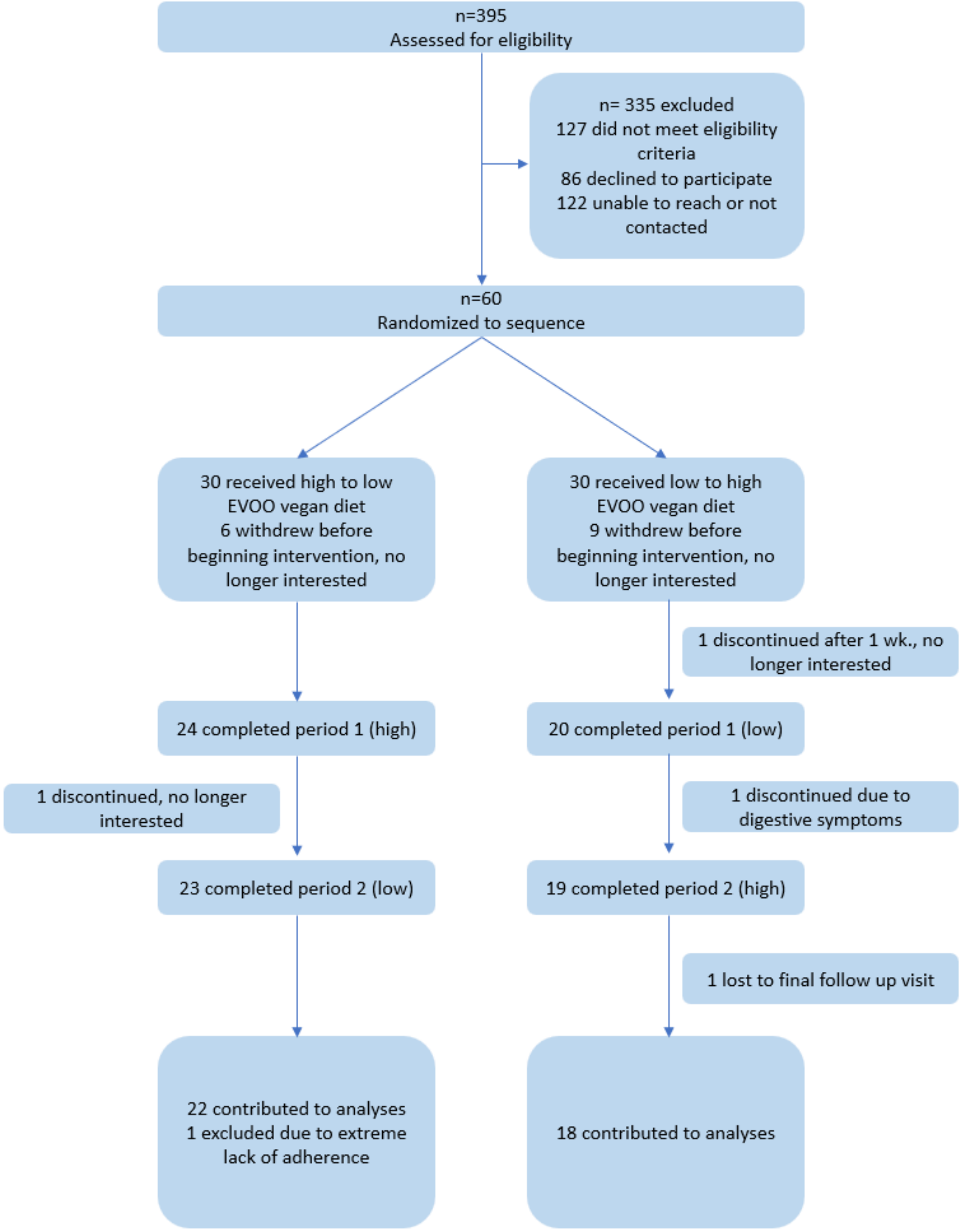

Figure adapted from Krenek et al [1].

SUPPLEMENTAL MATERIAL REFERENCES

1. Krenek AM, Mathews A, Guo J, Courville AB, Pepine CJ, Chung ST, et al. Recipe for Heart Health: A Randomized Crossover Trial on Cardiometabolic Effects of Extra Virgin Olive Oil Within a Whole-Food Plant-Based Vegan Diet. J Am Heart Assoc [Internet]. 2024;13. Available from: https://www.ahajournals.org/doi/10.1161/JAHA.124.035034
